# The Kaposi's sarcoma-associated herpesvirus viral interleukin 6 gene affects metastasis and expression of B cell markers in a murine xenograft model

**DOI:** 10.1371/journal.pone.0204947

**Published:** 2018-09-28

**Authors:** R. Amy Fullwood, Gregory M. Low, Emily P. Chase, Meagan Grasley, Soren S. Beal, Ian M. McCrary, Christian W. Daniels, Kayleigh Ingersoll, Bradford K. Berges

**Affiliations:** Department of Microbiology and Molecular Biology, Brigham Young University, Provo, UT, United States of America; Wuhan University School of Life Science, CHINA

## Abstract

Kaposi’s sarcoma-associated herpesvirus (KSHV) is a cancer-causing virus in humans, primarily affecting AIDS patients. KSHV causes a range of cancers including Kaposi’s sarcoma, pleural effusion lymphoma and multicentric Castleman’s disease. Current methods available for treating these cancers are relatively ineffective, and new targets for therapy are needed. The KSHV viral homolog of interleukin-6 gene (vIL-6) may play a significant role in tumor development and may serve as a new anti-cancer target, but its role in tumor formation is only partially understood. Here, a novel animal model was used to study how vIL-6 affects tumor development. Highly immune-deficient Rag2^-/-^γc^-/-^ mice were transplanted with an immortalized human B cell line (BJAB) harboring either wild-type (WT) KSHV or a mutant strain lacking vIL-6 ΔvIL-6). Solid tumors developed and total tumor mass and the number of tumors were characterized. The vIL-6 gene had no significant impact on tumor mass, but significantly more tumors were detected when vIL-6 was present. Significant differences in expression of B cell markers in cells from extracted tumors were detected based upon the presence of vIL-6. B cell markers in tumor cells were also compared to the same cell type in culture, prior to xenotransplantation; B cell markers were mostly downregulated during tumor formation and these changes did not differ based upon the presence of vIL-6. The only marker that significantly increased in expression during tumor development was CD30. Tumor blood vessels were quantified to determine if more angiogenesis occurred with vIL-6-expressing virus, but there was no significant difference. These data indicate that vIL-6 plays a role in KSHV tumor formation in B cells *in vivo*. Further investigation into how vIL-6 manipulates CD30 expression may shed insight into KSHV oncogenesis, and may identify how vIL-6 can be targeted.

## Introduction

Kaposi’s sarcoma herpesvirus (KSHV), also known as human herpesvirus 8, is a gammaherpesvirus associated with multiple types of cancers in highly immunocompromised individuals [1]. Kaposi’s Sarcoma (KS) was first diagnosed as a slow-growing cancer of endothelial cells, but a more aggressive form of KS was identified in homosexual populations during the early years of the Acquired Immunodeficiency Syndrome (AIDS) pandemic. KSHV itself was discovered in KS legions in 1994 [2].

KSHV infects B cells and is associated with persistent infections which can lead to lymphoma, especially in AIDS patients. Besides KS, KSHV is also thought to play a role in the development of B cell cancers such as primary effusion lymphoma (PEL) and Multicentric Castleman’s disease (MCD) [3]. Treatments for these cancers typically involve the use of monoclonal antibodies to target B cells for destruction, but the treatment also targets non-cancerous B cells [4]. New treatments are needed, since patient prognosis is typically poor for these cancers.

PEL is a malignant effusion of monoclonal B cells within the peritoneal, pleural, or pericardial space, and PEL is highly correlated with KSHV infection. KSHV infection in PEL is mainly latent with 2–5% of cells actively expressing vIL-6. These lymphomas usually do not form a solid tumor mass, and are typically found in HIV-infected individuals. PEL cells usually express CD45, CD30, CD38, and CD138, but lack the common B cell makers CD19 and CD20 [5]. PEL lymphomas make up 3% of all AIDS-related lymphomas, and effective treatments are lacking [6]. MCD is characterized by localized B cell proliferation and vascular proliferation, usually forming a solid tumor mass. MCD is typically seen in AIDS patients or transplant recipients, suggesting that immunosuppression is required. 5–25% of infected cells express vIL-6 in MCD tumors and generally appear to have a higher number of KSHV productively-infected KSHV cells than in PEL [3].

The mechanism by which KSHV causes these cancers is poorly understood. KSHV has pirated various primate/human genes, many of which encode immunomodulatory functions, and these may play important roles in viral persistence as well as oncogenesis [7, 8]. One of these genes is a homolog of human interleukin 6 (IL-6) called viral interleukin 6 (vIL-6). IL-6 serves as a pro-inflammatory cytokine [9], gp130 signal enhancer [10], a stimulator of angiogenesis [11, 12], a B cell proliferator [13], a factor involved in evasion of interferon responses [13], and a negative regulator of apoptosis [14]. vIL-6 shares ~25% amino acid identity with human IL-6 [15], and appears to share some functionality with IL-6 while also showing some differences. vIL-6/gp130 binding is ~1000-fold lower than the IL-6/IL-6R binding complex, making it less efficient as an extracellular cytokine than IL-6 [16]. IL-6 stimulates gp130 signaling through the IL-6R complex, whereas vIL-6 is able to directly bind gp130 and promote STAT/MAPK signaling without the need for IL-6R. vIL-6 is retained in cells to a greater extent than human IL-6, suggesting that vIL-6 acts more as an intracellular signaling molecule as compared to IL-6 [10].

vIL-6 also shares some properties with IL-6, such as the enhancement of B cell proliferation, the production of Vascular Endothelial Growth Factor (VEGF), and other cytokines (e.g., IL-10 and IL-12) [11, 16–18]. Transgenic expression of vIL-6 in mice results in hyperplastic germinal centers in the lymph nodes, plasmacytosis in the spleen, and other MCD-like phenotypes. Interestingly, vIL-6 transgenic mice lacking murine IL-6 did not manifest these phenotypes, suggesting that mammalian IL-6 and vIL-6 work together during oncogenesis [19].

To date, there is no established animal model for KSHV infection that can be used to explore the functions of vIL-6 in oncogenesis. Injection of KSHV-infected BCBL-1 cells into immunodeficient SCID mice resulted in tumor formation and angiogenesis into the tumor tissue of past research models [20]. These results suggest that the signals for angiogenesis emanating from KSHV tumors are compatible with the recruitment of murine blood vessels to the tumor.

Here, we transplant immortalized human B cells (BJAB) containing KSHV genomes into highly immunocompromised Rag2^-/-^γc^-/-^ mice as a model to understand how vIL-6 affects tumor growth. A mutant KSHV strain that lacks the vIL-6 gene is used [21], as well as a wild-type control where vIL-6 is intact. Our hypothesis is that vIL-6 will promote development of tumors, increase the tumor, and promote angiogenesis. By comparing tumors induced by KSHV with and without vIL-6 we hope to identify whether vIL-6 could be an effective target for treating KSHV-induced cancers.

## Materials and methods

### Cells and cell culture

Virus and cells for these experiments (BJAB KSHV wild-type (WT), BJAB ΔvIL6) were obtained from Dr. Lagunoff at the University of Washington [21]. The cells were then grown in RPMI 1640 containing 10% fetal bovine serum (FBS), penicillin/streptomycin, and 2mM L-glutamine. Cells were grown in 10μg/ml puromycin for both virus-infected cell lines and passaged for one month to select for puromycin-resistant/GFP+ cells. We maintained healthy stocks of BJAB with WT KSHV, and BJAB with a KSHV ΔvIL6 cells until ≥80% of virally-infected cells were GFP+ (as confirmed using flow cytometry).

### Development of tumors in mice

It had been previously shown that 5x10^6^ infected B cells cause visible tumors via subcutaneous injection in 2 out of 3 SCID mice [20]. We decided to use the higher immune-deficient Balb/c Rag2^-/-^γc^-/-^ mouse strain in order to promote a greater degree of tumor development. 5x10^6^ live cells from each group were suspended in 40μL of serum-free Iscove’s Modified Dulbecco’s Medium. 8–15 week old mice were injected subcutaneously over the abdomen with a 28 gauge needle. The needle remained in place for 30 seconds after injection, and was slowly removed. An initial 6 mice per study group were used, 3 male and 3 female; the experiment was then repeated, resulting in 12 total mice per study group. Animals were monitored daily for changes in health for 5 weeks. After 5 weeks tumors were palpable and the animals were sacrificed consistent with recommendations from the Panel on Euthanasia of the American Veterinary Medical Association. Mice were dissected and the tumors excised from the abdominal region (no tumors were detected in any other area). Total number of tumors, as well as individual tumor and total tumor mass per animal were measured and recorded. Single cell suspensions were made a filtered through a 40μm cell strainer. A 10% increase in the body mass of an animal during tumor formation or detection of tumors with a diameter greater than 1.5cm would have resulted in euthanasia, but no animals reached these values prior to sacrifice. No animals were found dead during these studies.

### RT-PCR analysis of vIL-6 mRNA expression

Total RNA was extracted from ~1mg of frozen tumor tissues using Trizol Reagent (ThermoFisher). RNA was treated with RNAse-free DNAse (New England Biolabs) and DNase was inactivated. cDNA was generated using the High Capacity cDNA Reverse Transcription kit (ThermoFisher) with random primers. Primers specific for the KSHV vIL-6 gene were then used for PCR amplification (For 5’ TGGAGCTTCTGACGAAGACCT 3’ and Rev 5’ AGTCCCTGAAGCCTCCCTAAT 3’), resulting in a 118bp product which was resolved on a 2% LE agarose gel. A plasmid containing the wild-type vIL-6 sequence was used as a positive control, and as negative controls tumor RNA from 6 animals infected with the ΔvIL-6 virus were used.

### Flow cytometric analysis

200,000 live cells were suspended in 100μL of FACS stain buffer and 3μL of Human/Mouse Fc block added [22] and incubated at 4°C for 15 minutes. 3μL of each desired antibody was added and incubated for 30 minutes at 4°C in the dark. Cells were fixed by adding 1mL 1% paraformaldehyde (in 1x PBS). Cells were then spun down for 3 minutes at 3,000rpm and suspended in 500μL of 1x PBS. The first staining panel included CD45 (PE-Cy7), CD19 (APC-eFluor780), CD20 (APC), CD138 (PE-Cy5.5) and CD22 (PE). The second staining panel included CD45 (FITC), CD19 (PerCP), CD38 (PE-Cy7) and CD30 (APC). Gating was determined using unstained populations run under the same voltage and compensation parameters, or in the case of GFP the use of non GFP-expressing BJAB cells. Propidium iodide and unstained controls of both cell types were run. BD Attune and Attune Cytometric Software Version 2.1 were used to run samples and obtain data.

### Quantification of tumor blood vessels

Tumors were frozen in OCT and then sectioned to a thickness of 12μm. Sections were stained with hematoxylin and eosin in order to visualize blood vessels within tumors. Blood vessels were counted and tumor size measured, and then blood vessels per square millimeter were calculated.

### Statistical analysis

Pooled t tests were performed in order to determine if significant differences existed between samples.

### Ethics approval and consent to participate

This study was carried out in strict accordance with the recommendations in the Guide for the Care and Use of Laboratory Animals of the National Institutes of Health. Approval to perform this research on mice was grant by the Brigham Young University Institutional Animal Care and Use Committee (protocol 150108).

## Results

### Analysis of number of tumors and total tumor mass

**Fig 1:**
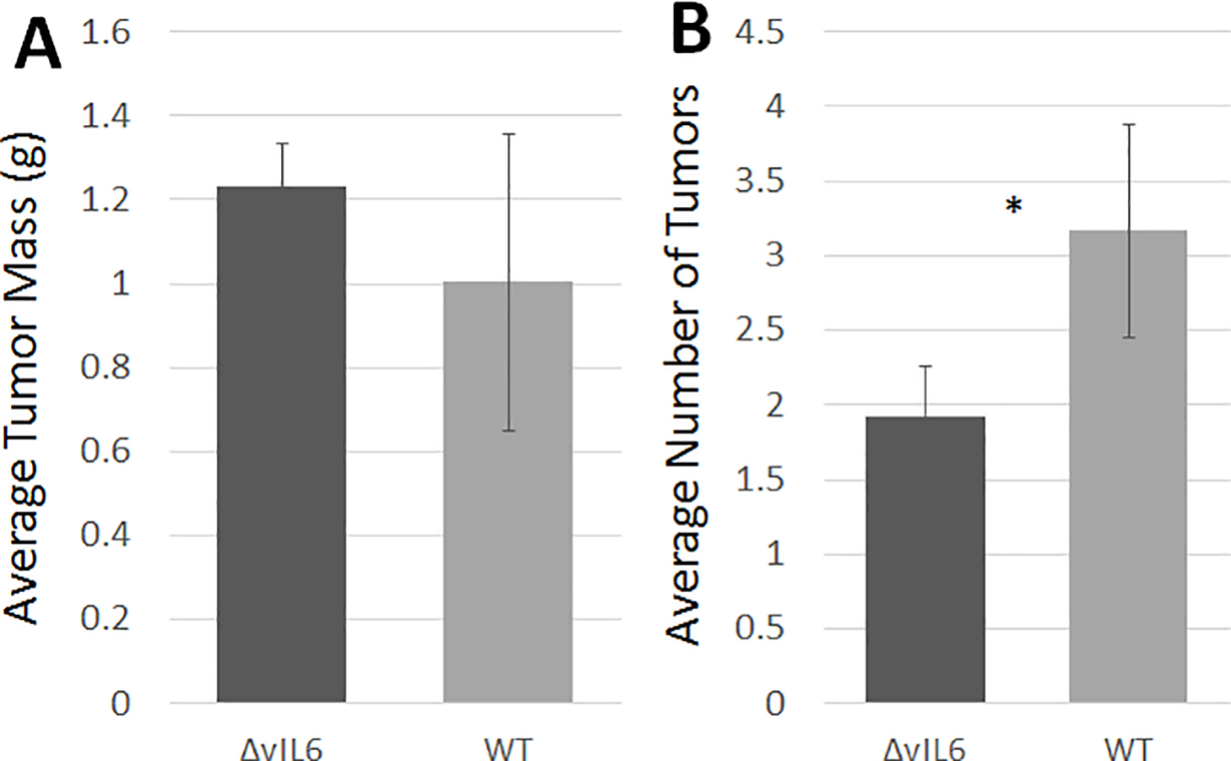
Measurement of tumor mass and number of tumors. Humanized mice were inoculated intraperitoneally with BJAB cells containing either WT KSHV or the ΔvIL6 strain. After 5 weeks, tumors were palpable above the abdomen, and animals were sacrificed and tumors were removed via dissection. (A) Measurement of tumor mass. Tumors showed no significant difference in mass between the infected WT and ΔvIL6 virus strains (p = 0.36). (B) Average number of tumors. Mice inoculated with BJAB cells containing the WT virus had significantly more tumors than those inoculated with the ΔvIL6 strain (p = 0.029). n = 11 WT; n = 12 ΔvIL-6. *P < 0.05.

BJAB cells harboring either wild-type (WT) KSHV or the ΔvIL-6- mutant (ΔvIL-6- mutant) were injected intraperitoneally into Rag2^-/-^γc^-/-^ mice, and after five weeks tumors were visible. Rag2^-/-^γc^-/-^ mice lack mature T cells and B cells due to the lack of the Rag2 protein (involved in recombination events necessary to generate T cell and B cell receptors), and they also lack mature natural killer (NK) cells due to the lack of the common gamma chain (γc) receptor which is involved in NK cell maturation. Thus, this mouse strain lacks an adaptive immune system, but most components of the innate immune system are intact. Mice were sacrificed and the number of tumors in the peritoneal cavity was counted; additionally tumors were dissected and the mass determined. There was no significant difference was detected in the mean tumor mass in animals injected with cells containing either WT KSHV (1.00g) or the ΔvIL-6 mutant (1.23g; p = 0.36; see Fig 1A). However, mice injected with WT KSHV cells had significantly more tumors than those injected with ΔvIL-6 cells (3.17 vs 1.92 tumors; p = 0.029; see Fig 1B).

### Comparison of WT KSHV and ΔvIL-6 cells extracted from tumors

**Fig 2:**
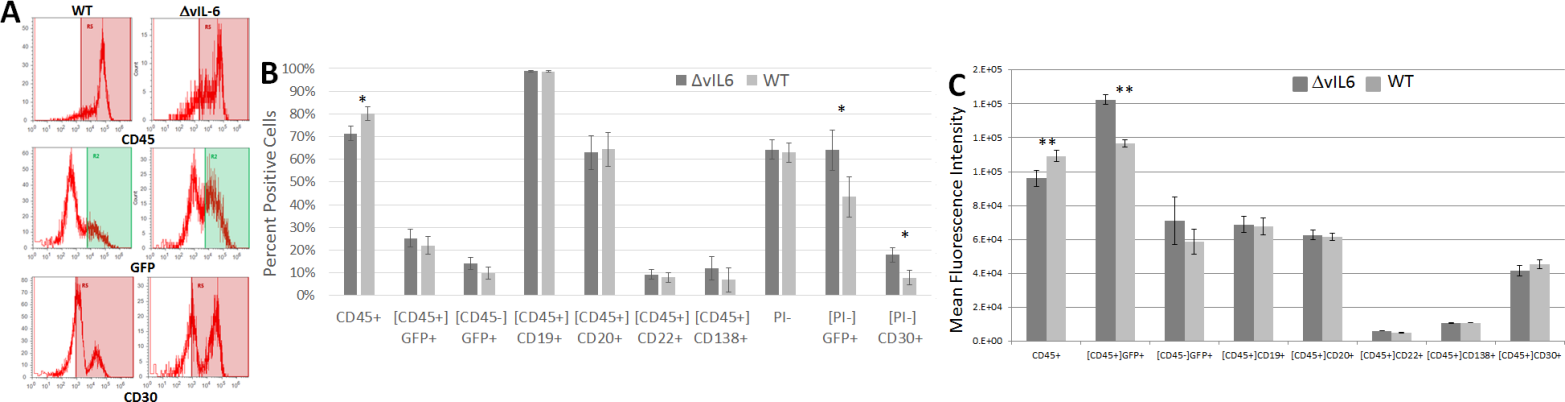
Analysis of B cell markers on cells from extracted tumors. Cells from either WT virus or ΔvIL-6 tumors were stained with antibodies specific for human B cells markers and analyzed by flow cytometry. The gating strategy is indicated by brackets, where [CD45] indicates that only human CD45+ cells were analyzed for the secondary marker shown below. (A) Representative histograms of some markers that differed by virus strain. (B) Summary of mean populations that were positive for the given marker. (C) Summary of mean fluorescence intensities. P values are indicated. n = 5 WT; n = 6 ΔvIL-6.

Following preliminary analysis of tumors, single cell suspensions were made and tumor cells were analyzed by flow cytometry to determine if the presence of vIL-6 influenced gene expression of common B cell markers on tumor cells (CD45, CD19, CD20, CD22, CD30, and CD138). These markers were examined because they are known to be expressed differently in primary B cells as compared to B cells immortalized by KSHV [3]. Both WT KSHV and ΔvIL-6 express GFP, allowing for rapid detection of infected cells and propidium iodide was used to exclude dead cells. Fig 2A shows the frequency of detection of the various B cell markers in tumor cells infected with either WT KSHV or ΔvIL-6 from representative animals, and a summary of the mean results in shown in Fig 2B. ΔvIL-6-infected tumor showed increased levels of GFP+ (25.12% in ΔvIL-6 and 21.98% in WT) and CD30+ (37.53% ΔvIL-6 and 15.35% in WT) cells compared to WT KSHV infection (p = 0.044 and 0.011, respectively). WT KSHV-infected tumor cells showed a higher frequency of CD45 expression compared with ΔvIL-6-infected tumor cells (p = 0.021). Mean fluorescence intensity (MFI) measures the brightness of cell markers on individual cells, and allows for quantification of specific protein expression by flow cytometry. An analysis of the MFI of these populations (Fig 2C) only showed significant differences in expression of CD45 (higher expression in WT KSHV-infected cells) and in GFP expression (higher expression in ΔvIL-6-infected cells). None of the other B cell markers showed any significant differences in MFI.

### Comparison of WT KSHV and ΔvIL-6 cells grown in culture

**Fig 3:**
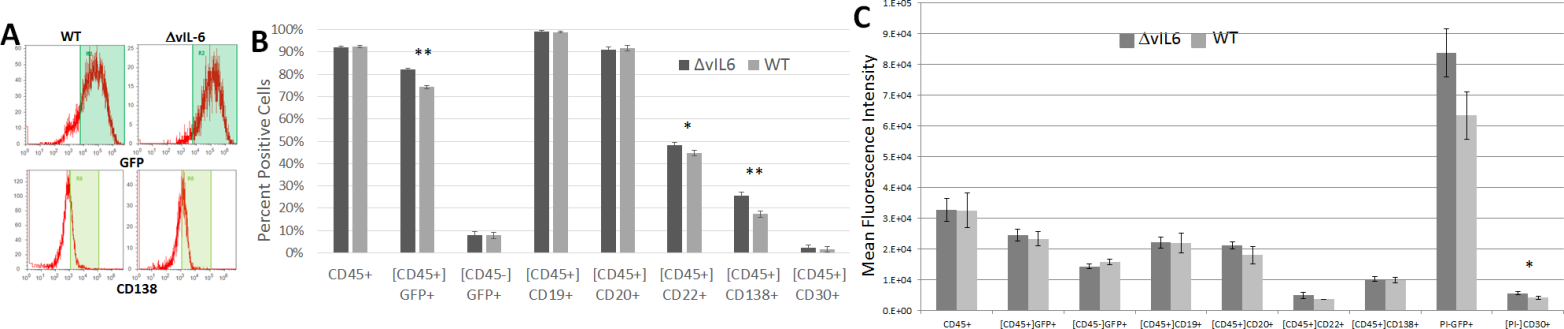
Analysis of B cell markers on BJAB cells grown in culture. BJAB cells containing either WT virus or the ΔvIL-6 strain were stained with antibodies specific for human B cells markers and analyzed by flow cytometry. The gating strategy is indicated by brackets, where [CD45] indicates that only human CD45+ cells were analyzed for the secondary marker shown below. (A) Representative histograms of some markers that differed by virus strain. (B) Summary of mean populations that were positive for the given marker. (C) Summary of mean fluorescence intensities. P values are indicated. n = 5 WT; n = 6 ΔvIL-6.

After determining that some cell surface expression markers differed in WT KSHV and ΔvIL-6 infected BJAB cells, we then examined these same markers on these same cell types grown in culture, and without tumor development *in vivo* (Fig 3). Fig 3A shows the frequency of detection of the various B cell markers in cultured cells infected with either WT KSHV or ΔvIL-6 from representative cell cultures, and a summary of the mean results in shown in Fig 3B. Both CD22 and CD138 were found to be more highly expressed in ΔvIL-6-infected cells as compared to WT-infected cells (p = 0.0098 and p = 0.0002, respectively). Additionally, the fraction of cells in culture that expressed GFP was also significantly higher in the ΔvIL-6 infected cells (p<0.0001). All other cell markers were very similar between the two cell types. An analysis of the MFI of these populations (Fig 3C) showed a lower intensity of CD30 expression in WT KSHV-infected tumor cells compared with ΔvIL-6-infected tumor cells (p = 0.044). None of the other B cell markers showed any significant differences in MFI.

### vIL-6 is expressed in solid tumors

It was previously reported that vIL-6 mRNA is not detectable from the WT construct in latently-infected BJAB cells (it is considered a predominantly lytic-phase gene), but that it could be detected by northern blot if the virus was induced to replicate [23]. We wanted to confirm if vIL-6 was expressed in our *in vivo* model. We performed RT-PCR on RNA extracted from both WT and ΔvIL-6 BJAB cell tumors using a primer set directed towards the deleted sequence in vIL-6 (see Methods) and which is anticipated to produce a product of 118bp. We found detectable vIL-6 mRNA in 4 of the 5 wild-type tumor samples (results summarized in Table 1). The experiment was repeated beginning with RNA extraction and the same result was obtained (the same tumor sample had undetectable expression). We thus conclude that vIL-6 expression is common in tumors in our model, but that it is not detectable in all tumors. Our results are similar to a previously published report using SCID mice engrafted with KSHV+ BCBL-1 cells, where lytic KSHV gene expression, including vIL-6 expression, was detected in solid tumors [20].

**Table 1:** Analysis of tumors for vIL-6 mRNA expression and measurement of angiogenesis in tumors.

Virus strain	Mouse	Tumors detected	# tumors analyzed	vIL-6 mRNA detection	Blood vessels/mm^2^
KSHV WT	A	0	0	n/a	n/a
	B	2	2	yes	0
	C	7	5	no	0
	D	5	4	yes	0.767
	E	8	3	yes	0.018
	F	4	2	yes	0
KSHV ΔvIL-6	A	4	3	no	0.108
	B	2	1	no	0.945
	C	1	1	no	0.432
	D	3	3	no	0.635
	E	4	3	no	0.552
	F	2	2	no	0.015

Tumors from 5 WT and 6 ΔvIL-6 mice were prepared for histology by freezing in OCT compound and stored at -80°C, then sectioned to a thickness of 12μm. RNA was extracted from 5 slides and subjected to RT-PCR using a primer set for the vIL-6 mRNA. Other sections were stained with hematoxylin and eosin and analyzed for the presence of blood vessels, which were then correlated to the area of the tumor.

### Analysis of changes in gene expression patterns during the process of tumor development

**Fig 4:**
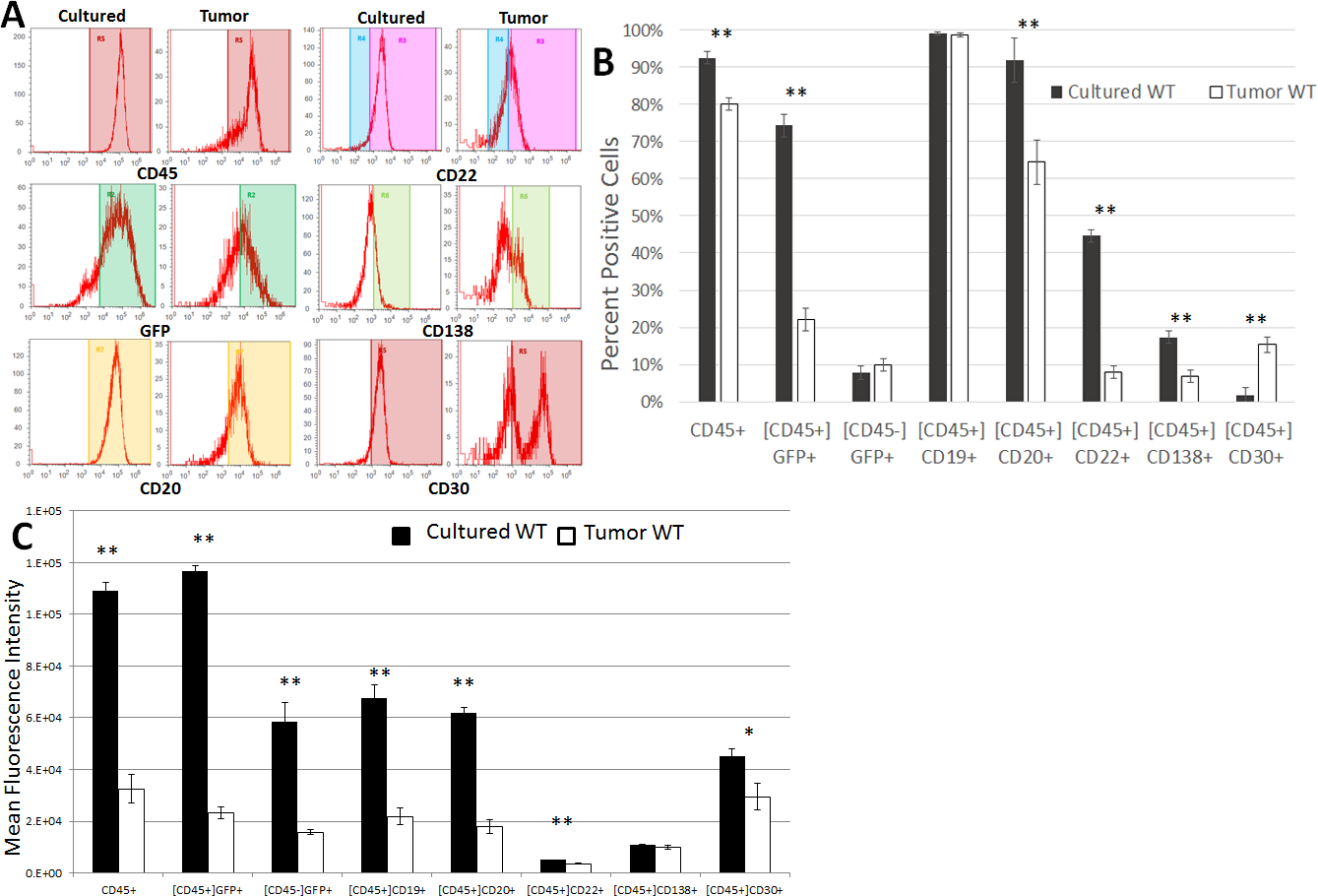
Changes in gene expression patterns during tumor development in WT-infected cells. BJAB WT cells from culture and single cell suspensions from solid tumors were stained for common B cell markers and analyzed by flow cytometry. B cell markers were analyzed from these different cellular populations to determine if changes in gene expression were evident during the process of tumor development. Populations were gated on the human CD45 marker as indicated by brackets, then various B cell markers were analyzed. (A) Representative histograms of some B cell markers that differed in cultured cells vs tumor cells. (B) Summary of mean populations that were positive for the given marker. (C) Summary of mean fluorescence intensities. n = 5 WT; n = 6 ΔvIL-6. Standard errors are indicated; **, p≤0.01.

A more detailed analysis of the expression profiles (six different cell surface markers, plus GFP expression to indicate viral infection) of either cultured cells or tumor cells from the above experiments was then performed in order to better determine what changes in gene expression took place during the process of tumor development (Fig 4). Results from representative cell cultures or solid tumors are shown in Fig 4A, while mean results are shown in Fig 4B. In WT-infected cells, significant downregulation of the following markers occurred during tumor development (Fig 4A): CD45+ (92.5% to 80.1% in culture vs in tumors, respectively; p<0.0001), CD45+GFP+ (74.31% to 21.98%; p<0.0001), CD20+ (91.81% to 64.4%; p = 0.0009), CD22+ (44.61% to 8.05%; p<0.0001), and CD138+ (17.33% to 6.86%; p = 0.0001). We noted that the CD138+ cells formed a single major population in culture, but after tumor formation both higher and lower expressing populations emerged. Interestingly, the expression of CD30 was significantly upregulated during WT tumor development (1.69% to 15.35%; p<0.0001).

When the MFI was analyzed for each of these markers in WT virus-infected cells (Fig 4C), we found that the results were mostly similar to those seen when gating on the positive populations, as above. For example, CD45+, GFP+, CD20+, and CD22+ cells decreased in both total numbers (Fig 4B) and also in expression levels (Fig 4C). However, some discrepancies were also noted. The CD45-GFP+ population was not significantly different in the percentages of cells when comparing them from cultured cells to tumor cells, but the MFI for GFP in this population was significantly reduced during the process of tumor formation (p = 0.0004). That same observation was made for CD19 during tumor formation; essentially all cultured cells and tumor cells expressed CD19 (not significant; p = 0.503) but the MFI for CD19 was significantly higher in cultured cells vs. tumor cells (p<0.0001; Fig 4C). CD138 was significantly downregulated (p = 0.0001) during tumor formation in terms of the numbers of positive cells, but there was no significant difference in the MFI between these populations (p = 0.145). Interestingly, the percentage of cells expressing CD30 significantly increased in cells infected with the WT virus during the process of tumor formation (Fig 4B), but the MFI was significantly decreased in these cells (Fig 4C).

**Fig 5:**
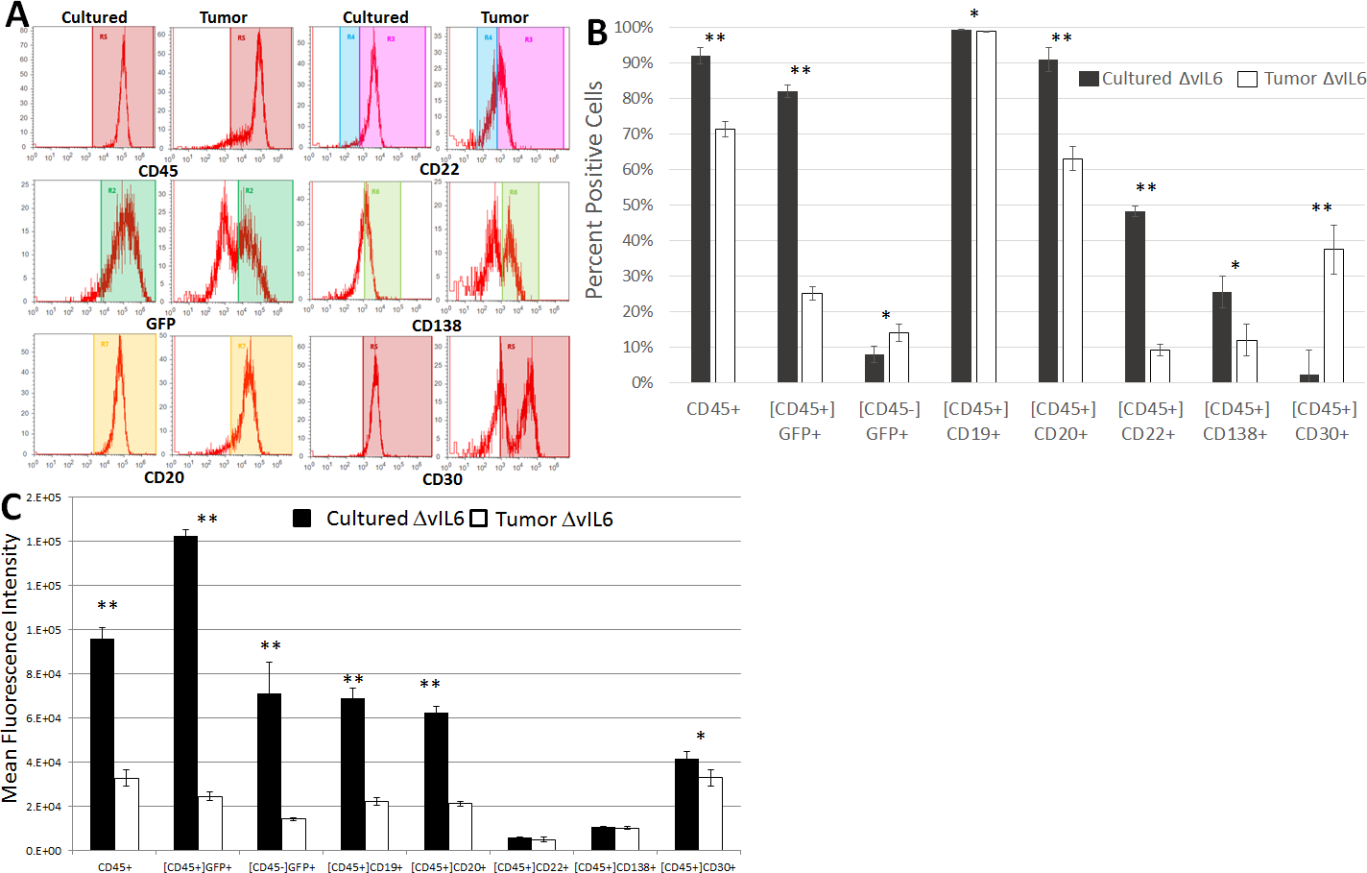
Changes in gene expression patterns during tumor development in ΔvIL6-infected cells. BJAB ΔvIL6 cells from culture and single cell suspensions from solid tumors were stained for common B cell markers and analyzed for flow cytometry. B cell markers were analyzed from these different cellular populations to determine if changes in gene expression were evident during the process of tumor development. Populations were gated on the human CD45 marker as indicated by brackets, then various B cell markers were analyzed. (A) Representative histograms of some B cell markers that differed in cultured cells vs tumor cells. (B) Summary of mean populations that were positive for the given marker. (C) Summary of mean fluorescence intensities. n = 5 WT; n = 6 ΔvIL-6. Standard errors are indicated; *, p≤0.05 **, p≤0.01.

Similarly, ΔvIL-6-infected cell markers from cultured cells and cells extracted from tumors were analyzed to determine differences in gene expression patterns during tumor development (Fig 5). Results from representative cell cultures or solid tumors are shown in Fig 5A, while mean results are shown in Fig 5B. The following markers were downregulated during tumor development: CD45+ (92.03% to 71.38% in culture vs in tumors, respectively; p<0.0001), CD45+GFP+ (82.04% to 25.12%; p<0.0001), CD19+ (99.3% to 98.9%; p = 0.012), CD20+ (91.0% to 63.1%; p<0.0001), CD22+ (48.24% to 9.21%; p<0.0001), and CD138+ (25.52% to 12.0%; p = 0.011). As noted for WT-infected cells, the CD138+ cells formed a single major population in culture, but after tumor formation both higher and lower expressing populations emerged. The CD45-GFP+ (8.0% to 14.06%; p = 0.024) and CD30+ (2.25% to 37.53%; p = 0.003) populations significantly increased. When the MFI was analyzed for each of these markers (Fig 5C), we found that many cellular populations were in accordance between the percentage of cells positive for the marker changing along with the MFI (e.g., CD45+, GFP+, CD19+, CD20+ all decreased by both parameters during tumor formation). Interestingly, the CD45-GFP+ population was significantly increased in terms of the percentage of cells during tumor formation (p = 0.024), while the MFI actually dropped during tumor formation (p = 0.0033). While CD22 and CD138 both decreased in the number of positive cells during tumor formation, there was no significant difference in MFI noted (p = 0.16 and 0.32, respectively). Similar to what was seen in WT-infected cells, significantly more ΔvIL-6-infected tumor cells expressed CD30 as compared to cultured cells, but the MFI was significantly lower in tumor cells (p = 0.045).

### Measurement of blood vessels in solid tumors

Angiogenesis, or the recruitment of new blood vessels is a common phenomenon in malignant tumors. Since vIL-6 is proposed to promote angiogenesis via VEGF [11], we analyzed the number of blood vessels per tumor area in tissue sections by hematoxylin and eosin staining. We found a high degree of variability in the number of blood vessels per tumor, with 0.45+/-0.14 vessels/mm^2^ for ΔvIL-6 tumors (range of 0.015 to 0.945) and 0.16 = /-0.15 vessels/mm^2^ for WT tumors (range of 0 to 0.767). Results are summarized in Table 1. No significant difference in blood vessels per tumor area was detected by virus type (p = 0.20; two sample unequal variance t test).

## Discussion

In this study, immortalized human B cells infected with recombinant KSHV were injected into highly immunodeficient mice and solid tumors developed. In order to better understand the role played by vIL-6 in tumor formation, a mutant strain with the vIL-6 gene deleted was used and compared to WT KSHV. Solid tumors formed with both cell types, and no significant difference in tumor mass was noted whether vIL-6 was present or not. However, there was a significant difference in the number of tumors found (p = 0.029), with fewer tumors detected in animals injected with cells infected with the ΔvIL-6 strain. A number of B cell surface markers were analyzed by flow cytometry, both in immortalized cells in culture as well as in cells derived from solid tumors, to determine any impacts of vIL-6 expression on host cell gene expression.

BJAB cells mostly expressed CD45, CD19, and CD20 in culture, while only ~50% expressed CD22 and ~20% expressed CD138, regardless of the presence of the vIL-6 gene. CD30 expression was very low in cultured cells, but rose during tumor formation (and preferentially in ΔvIL-6 cells). By way of comparison, PEL cells typically express the pan-leukocyte marker CD45 [5], as well as the activation/immunoblastic marker CD30 [24]. PEL cells also commonly express the plasma cell marker CD138, but lack the pan-B cell marker CD19 and CD20 [24], which may regulate B cell activation and proliferation. Thus, KSHV+ BJAB cells in our model showed some similarities with the typical PEL phenotype with the major differences being that CD19 and CD20 are not typically expressed in PEL cells, but in culture our BJAB cells commonly expressed these markers. Upon solid tumor formation, CD19 expression decreased in MFI measurements and CD20 decreased in both the percentage of positive cells and in MFI. During the process of tumor formation, BJAB cells showed a slight reduction in CD45 expression (about 15–20% cells), continued to express CD19, and reduced CD20 expression in about 30% of cells. CD22 expression increased in about 20% of cells and CD138 increased in about 30% of cells.

We expected to see a greater tumor mass and larger number of tumors with WT virus as compared to ΔvIL-6, since vIL-6 is thought to play a role in angiogenesis *in vivo* and thus could provide additional oxygen and nutrients to a growing tumor. However, no significant difference in tumor mass was detected based upon the presence of absence of vIL-6. However, a significant difference in the total number of tumors was found with fewer tumors present with the ΔvIL-6 virus. These results suggest that vIL-6 may contribute to the formation of metastases. The spread of tumor cells can be mediated by the presence of blood vessels, and so an angiogenic function of vIL-6 could potentially explain this difference. However, we analyzed the number of blood vessels per area in tumors containing either the WT or the ΔvIL-6 strains and did not find a significant difference.

Interestingly, a 2015 report showed that vIL-6 can upregulate cellular genes involved in migration, resulting in enhanced endothelial cell movement. Specifically, CEACAM1 was upregulated and this protein was required for the increased cellular migration [18]. While the present study analyzed B cell tumors and not endothelial cells, KSHV causes cancer in both cell types and vIL-6 could induce similar pathways in both cell types. Indeed, in the mentioned study they showed that CEACAM1 expression was also modulated in vIL-6-expressing human umbilical vein endothelial cells and also BCBL-1 cells, a B cell line [18].

When tumor cells were extracted and analyzed by flow cytometry, we found that ΔvIL-6 cells expressed CD30 to a significantly higher extent than WT cells. Analysis of the MFI of CD30 expression also revealed that the expression levels in just the positive cells were also higher in the ΔvIL-6 population. CD30 expression is normally seen in activated, but not resting B cells [25], suggesting that vIL-6 may shift B cells away from an activated phenotype during tumorigenesis. CD30 has a pro-apoptotic activity in some B cell cancers but not in others [26, 27], and cells depleted of vIL-6 through siRNA targeting showed higher levels of apoptosis [10]. These results suggest that vIL-6 may protect B cells from apoptosis during tumorigenesis via a pathway that involves CD30 expression. We also found that WT cells expressed significantly more CD45 than ΔvIL-6 cells. CD45 helps to regulate cell growth and oncogenic transformation in B cells [28], suggesting that the vIL-6 gene plays a role in tumorigenesis and possibly via a CD45-related pathway.

After discovering that WT and ΔvIL-6 tumor cells showed differences in gene expression patterns, we analyzed these same cell types in culture and without ever having formed a tumor to determine if those differences existed prior to tumor formation or if the changes occurred during tumor formation. Interestingly, the cell markers that differed in cell culture conditions were somewhat different than those detected in tumor cells; CD22 and CD138 were more commonly expressed in ΔvIL-6 cells in culture but not in tumor cells. We noted that CD22 and CD138 expression was not changed by MFI measurement (Fig 5C), suggesting that while some cells downregulated expression of these markers during tumor development it was on a per cell basis, and not a phenomenon that globally applied to the entire cell population. CD45 was expressed at higher levels in WT tumor cells, but not in cultured cells, suggesting that CD45 upregulation only took place in cells forming a solid tumor and was not intrinsic to cultured cells containing the vIL-6 gene. GFP expression from the recombinant KSHV genome was significantly higher in live ΔvIL-6 cells in culture, but this was not seen in tumor cells, even though both cell types were maintained in puromycin selective media for the at least 2 months prior to this analysis. These findings may suggest that gene expression differences may exist in the viral genome itself, possibly due to chromatin differences based upon the presence/absence of ΔvIL-6.

We then compared B cell marker expression in cultured cells to the same cell type after tumor development to determine how gene expression changed during the process of tumor development. 5 different markers were found to significantly decrease during the process of tumor development, and these changes were consistent for WT and ΔvIL-6 cells: CD45, CD45+GFP+, CD20, CD22, and CD138. These decreases may be indicative of a general de-differentiation of cells, which is a common feature in carcinogenesis. However, CD30 expression was found to be significantly increased in both WT and ΔvIL-6 cells, although ΔvIL-6 cells showed a greater increase. As mentioned above, CD30 can have pro-apoptotic activities and tumor cells may be undergoing apoptosis, which would explain the general upregulation of CD30.

However, 2 differences in gene expression were only noted in a single cell type when comparing WT to ΔvIL-6: CD19 expression was unchanged in WT cells but was downregulated in ΔvIL-6 cells, and the CD45-GFP+ population was unchanged in WT cells but was increased in ΔvIL-6 cells. The CD45-GFP+ population must represent virally-infected cells, because KSHV is not known to effectively infect murine cells, and so we believe that these cells have downregulated CD45 expression. As mentioned above, vIL-6 appears to have an ability to upregulate CD45 as a pro-growth function. These findings suggest that vIL-6 may prevent a downregulation and promote an upregulation in CD45 expression. CD19 also can play a role in B cell proliferation [29] and so its downregulation in ΔvIL-6 cells also aligns with this hypothesis. GFP expression was also found to be substantially decreased during the process of solid tumor formation. It is possible that the virus was lost from the BJAB cells, since puromycin selection ceased upon cell inoculation into animals. However, the KSHV LANA protein effectively promotes genome maintenance in infected cells and so it is not clear why GFP expression drops. It is also possible that epigenetic modifications of the GFP gene took place during the process of tumor formation, resulting in a silencing of the GFP gene.

## Conclusions

The presence of the KSHV vIL-6 gene has no impact on total tumor mass in a xenograft model, but there were more tumors when the vIL-6 gene was present. During the process of tumor development cells tended to downregulate expression of B cell markers in a generalized de-differentiation process that was common between both cell types. In contrast, a pro-apoptotic marker (CD30) was expressed at higher levels in cells lacking vIL-6 and some pro-growth markers (notably CD45) were upregulated cells with vIL-6 during tumor formation, suggesting that vIL-6 promotes cell growth and inhibition of apoptosis *in vivo* during solid tumor formation.

## Abbreviations

KSHV: Kaposi’s Sarcoma-associated Herpesvirus
IL-6: Interleukin-6
vIL-6: viral homolog of interleukin-6
KS: Kaposi’s Sarcoma
AIDS: Acquired Immunodeficiency Syndrome
PEL: primary effusion lymphoma
MCD: Multicentric Castleman’s disease
VEGF: Vascular Endothelial Growth Factor
